# Complete sequence of heterogenous-composition mitochondrial genome (*Brassica napus*) and its exogenous source

**DOI:** 10.1186/1471-2164-13-675

**Published:** 2012-11-28

**Authors:** Juan Wang, Jinjin Jiang, Xiaoming Li, Aimin Li, Yongtai Zhang, Rongzhan Guan, Youping Wang

**Affiliations:** 1 Jiangsu Provincial Key Laboratory of Crop Genetics and Physiology, Yangzhou University, Yangzhou, 225009, China; 2 Jiangsu Institute of Agricultural Science in the Lixiahe District, Yangzhou, 225009, China; 3 State Key Laboratory of Crop Genetics and Germplasm Enhancement, Nanjing Agricultural University, Nanjing, 210095, China

## Abstract

**Background:**

Unlike maternal inheritance of mitochondria in sexual reproduction, somatic hybrids follow no obvious pattern. The introgressed segment *orf138* from the mitochondrial genome of radish (*Raphanus sativus*) to its counterpart in rapeseed (*Brassica napus*) demonstrates that this inheritance mode derives from the cytoplasm of both parents. Sequencing of the complete mitochondrial genome of five species from *Brassica* family allowed the prediction of other extraneous sources of the cybrids from the radish parent, and the determination of their mitochondrial rearrangement.

**Results:**

We obtained the complete mitochondrial genome of *Ogura*-cms-cybrid (*oguC*) rapeseed. To date, this is the first time that a heterogeneously composed mitochondrial genome was sequenced. The 258,473 bp master circle constituted of 33 protein-coding genes, 3 rRNA sequences, and 23 tRNA sequences. This mitotype noticeably holds two copies of *atp9* and is devoid of *cox*2-2. Relative to *nap* mitochondrial genome, 40 point mutations were scattered in the 23 protein-coding genes. *atp6* even has an abnormal start locus whereas *tatC* has an abnormal end locus. The rearrangement of the 22 syntenic regions that comprised 80.11% of the genome was influenced by short repeats. A pair of large repeats (9731 bp) was responsible for the multipartite structure. Nine unique regions were detected when compared with other published *Brassica* mitochondrial genome sequences. We also found six homologous chloroplast segments (*Brassica napus*).

**Conclusions:**

The mitochondrial genome of *oguC* is quite divergent from *nap* and *pol*, which are more similar with each other. We analyzed the unique regions of every genome of the *Brassica* family, and found that very few segments were specific for these six mitotypes, especially *cam*, *jun,* and *ole*, which have no specific segments at all. Therefore, we conclude that the most specific regions of *oguC* possibly came from radish. Compared with the chloroplast genome, six identical regions were found in the seven mitochondrial genomes, which show that the *Brassica* family has a stable chloroplast-derived source.

## Background

The major function of the mitochondria (mt), as a semiautonomous organelle, in plant growth and development is to provide energy through oxidative phosphorylation [1]. In different to the small mt genome of animals (~16 kb), plants have longer mtDNA ranging from 200kb to 2000kb [2,3]. To date, several mt genomes from fertile and sterile plant species have been sequenced, including *Arabidopsis thaliana*[4], *Oryza sativa*[5-7], *Beta vulgaris*[8,9], *Zea mays*[10,11], *Nicotiana tabacum*[12], *Triticum aestivum*[13,14], and five species from the *Brassica* genus, i.e., *B. napus* (*pol*, *nap*), *B. rapa* (*cam*), *B. oleracea* (*ole*), *B. juncea* (*jun*), and *B. carinata* (*car*) [15-17]. The sequencing results indicated that apart from ribosomal protein genes, protein-coding genes are also relatively conserved both in nucleotide sequence and in number. However, the non-coding sequences are quite inconsistent among species, and even within the same species. The presence of large and short repeats is responsible for the dynamic multipartite structures, reorganization, and recombination [17].

In higher plants, mitochondrial inheritance usually follows the maternal origin during sexual hybridization. However, much more complicated modes are detected in somatic hybridization, wherein mt genome inheritance is derived from either or both biparents [18]. In the latter pattern, part of the mt genome, including cytoplasmic male sterility (CMS) genes, can be transferred from the donor parent to the receptor parent, and the introgressed segment experiences extensive rearrangement and recombination with the mtDNA of the receptor one. *Orf138*, originally identified in radish, was transferred successfully to various species, including *Arabidopsis*, *B. napus*, and *B. oleracea* by somatic hybridization [19-25].

*CMS* genes have a defect in the production of functional pollen. Generally, genes associated with *CMS* genes are located in the periphery of certain known mitochondrial genes and are cotranscribed with them [26]. *T-urf13* (*orf115*) was the first identified aberrant gene in the Texas (T)-cytoplasm of maize, which encodes a 13 kDa membrane-spanning polypeptide that depolarizes the mitochondria and leads to cell death [27-29]. In the BT (Boro II)-type CMS line of rice, *orf79* was cotranscribed with the *atp6* gene forming a 2.0 kb transcript [30]. The expressed protein contains a predicted transmembrane domain [31].

In the *Brassica* genus, the complete mt genomes of five species are sequenced, coupled with the basic feature of published *CMS* genes, which allows the detection of the extraneous source from donor parent (radish) of somatic hybrids.

## Results

### Genome size and nucleotide sequence in the genic region

**Figure 1:**
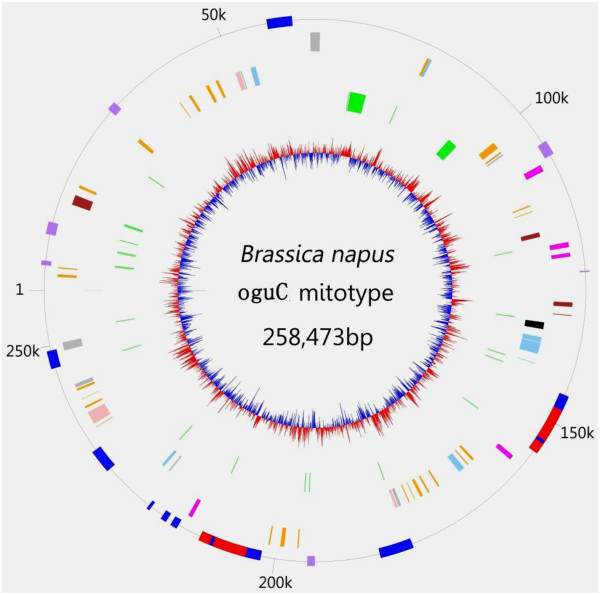
**Physical map of *oguC *mitochondria genome.** Circle display (from inside). First circle, the distribution of GC content. Second circle, tRNA and rRNA. Third circle and fourth circle, the genes transcribed clockwise and counterclockwise, respectively. The every color represents the gene group listed in the above box. Fifth circle, physical map scaled in kilobase pairs. Red, large repeat; Blue, unique region; purple, chloroplast-derived sequence.

**Figure 2:**
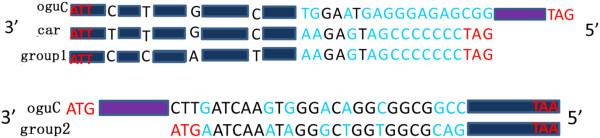
**The comparison of nucleotide sequence of *tatC *(A) and *atp6 *(B) from *Brassica *family.** Purple box means the extension segments in *oguC*, black box represents the identical segments rooted in the mtDNA of *Brassica* family. Group 1 contains *ole*, *cam*, *pol*, *jun* and *nap*. Group 2 represents *ole*, *cam*, *pol*, *jun*, *nap* and *car*.

The *oguC* mt genome was assembled into a 258,473 bp master circle with 45.21% G+C content (Figure 1). It encodes 33 proteins, three ribosomal RNA sequences (18s, 26s, and 5s), and 23 tRNA sequences, which account for 25.42% of the genome in total. Of these 33 protein-coding genes, two were identical copies of *atp9* and the *cox2-2* gene were absent. Taken mtDNA from *nap* CMS as control, two genes were detected to change the coding length. One of them is *tatC*, whose several continuous mutations were observed in the 3′-end and the stop codon was extended 27bp away (Figure 2A). Similar to the change in *tatC*, an additional 498 bp (including start codon) was placed in the 5′-end of *atp6*, whose nucleotide was completely identical to the one from radish (Figure 2B). Most of the other protein-coding genes were conserved in length, but the point mutation occurred extensively, where 40 single nucleotide polymorphisms (SNPs) were identified scattered among the 23 genes when compared to *nap*. Of the 40 SNPs, 13 were synonymous and 27 were non-synonymous (Table 1). Most of the variations were transitions. Compared with their counterparts in *Raphanus sativus, cox1*, *rps12*, and *atp8* were also the same in terms of amino acid and nucleotide sequence except for *atp6*. *ccmC* had 10 nucleotide substitutions, where 4 were non-synonymous. Using *R. sativus* as the control, only two SNPs were detected: one differs by a synonymous mutation in position 126, similar to the alignment of *nap*; the other was a G to A mutation in position 146, which caused a Thr to Gly switch. Among the 34 protein-coding genes in *pol*, 31 have an identical copy in *nap* and only 3 genes showed locus polymorphism [15]. Consequently, many variations in *oguC* may be associated with its background of somatic hybridization and complicated evolution.

**Table 1:** **SNP in protein-coding genes of mtDNA between ***oguC ***and *nap***

**Gene**	**Nucleic acid**^**a**^	**Amimo acid**^**b**^	**Mutation type**^**c**^	**SNP type**
*nad1*	571 C-T	191 L-F	N	transition
*nad2*	367 T-C	123 C-R	N	transition
*nad3*	265 T-C		S	transition
*nad4*	77 C-T	26 P-L	N	transition
	1205 C-T	402 P-L	N	transition
*nad7*	1079 T-C	360 F-S	N	transition
cox1*	109 C-T		S	transition
	111 T-A		S	transversion
	786 T-G		S	transversion
*cox2-1*	379 T-C	127 W-R	N	transition
*ccmB*	353 G-A		N	transition
ccmC**	126 A-G		S	transition
	155 G-A	52 R-H	N	transition
	338 G-A	113 R-K	N	transition
	351 G-A		S	transition
	476 C-A		S	transversion
	533 G-A	178 G-A	N	transition
	551 G-A	184 G-D	N	transition
*ccmFC*	283 G-A	95 E-K	N	transition
*ccmFN1*	361 T-A	121 F-I	N	transversion
	1000 C-T	334 L-F	N	transition
*ccmFN2*	380 C-T	127 L-S	N	trnasition
*atp1*	987 T-C		S	transition
*atp4*	176 C-T	59 A-V	N	transition
atp8*	370 A-C	124 L-I	N	transversion
	448 T-C	150 A-V	N	transition
*atp9*	64 A-G	22 I-V	N	transition
*rps3*	1254 A-C		S	transversion
	1320 A-C		S	transversion
*rps4*	189 T-G		S	transversion
	776 C-T	259 S-F	N	transition
rps12*	12 T-G	4 F-L	N	transversion
	336 A-C	112 R-S	N	transversion
	345 A-C		S	transversion
*rpl2*	464 G-A	155 G-D	N	transition
	840 C-T		S	transition
	1004 C-T	335 S-L	N	transition
*rpl5*	515 T-C	172 L-P	N	transition
*rpl16*	506 C-T	169 P-L	N	transition

^a^ Location of base mutation, ^b^Location and switch of amino acid mutation, ^c^ S,Synonymous; N, Non-synonymous.

* Nucleotide sequence of genes identical to *Raphanus sativus* ** Two SNPs when compared to *Raphanus sativus*.

### Reconstruction of the nap-CMS cybrid mitochondrial genome

**Figure 3:**
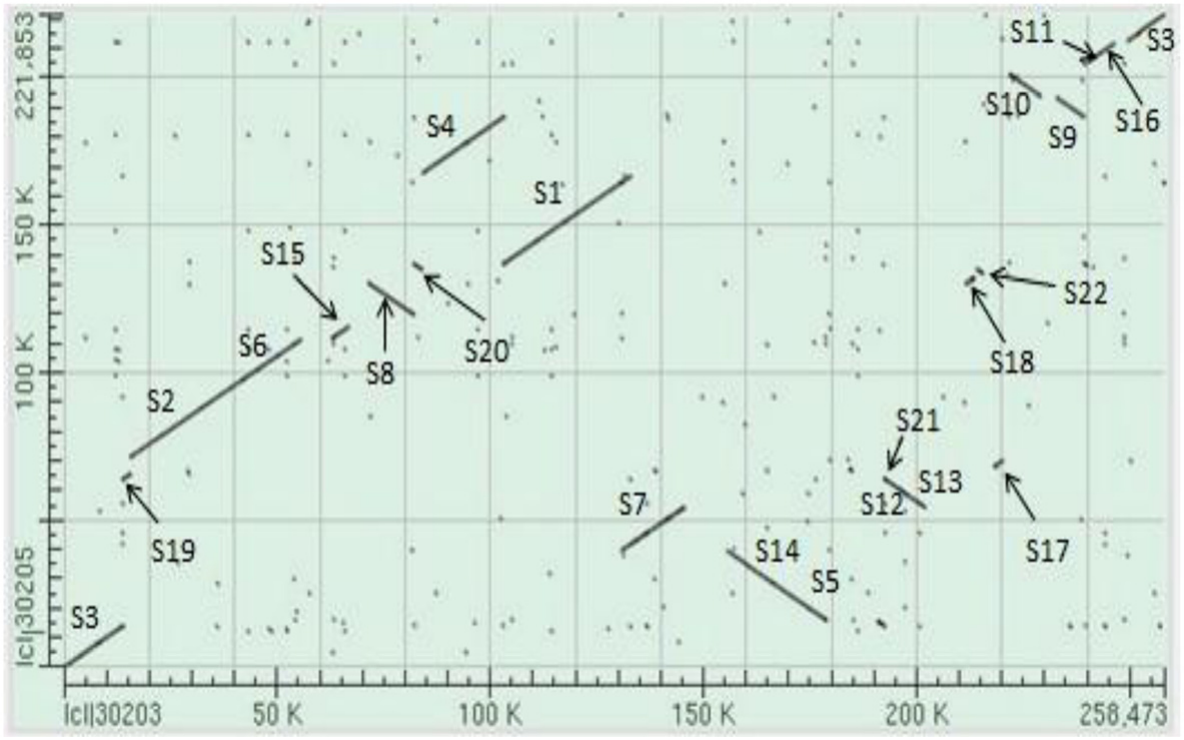
**Syntenic regions of *oguC *and *nap *mitotype genome.** S1-S24 refer to the syntenic regions (similarity ≥ 95%, length ≥1 kb). Horizontal axis and vertical axis mean the whole genome nucleotide sequence of *oguC* and *nap*, respectively.

The syntenic regions of *oguC* and *nap* were analyzed using a bl2seq algorithm. A total of 22 segments ranging from 1393 bp to 30232 bp possessed at least 95% similarity and at least 1 kb in size, which were responsible for 80.11% and 92.78% of these two mitotype genomes, respectively. The majority of the syntenic regions contained 99% similarity except S10 (97%), S16 (98%), S18 (96%), S20 (96%), and S21 (95%). The direction of 10 regions was identical, but that of the other 12 was the opposite (Figure 3). Estimating the minimum recombination events that occurred to account for the restructuring of the two mitotypes was difficult because of the many syntenic regions.

### Reorganization of the mitochondrial genome

**Figure 4:**
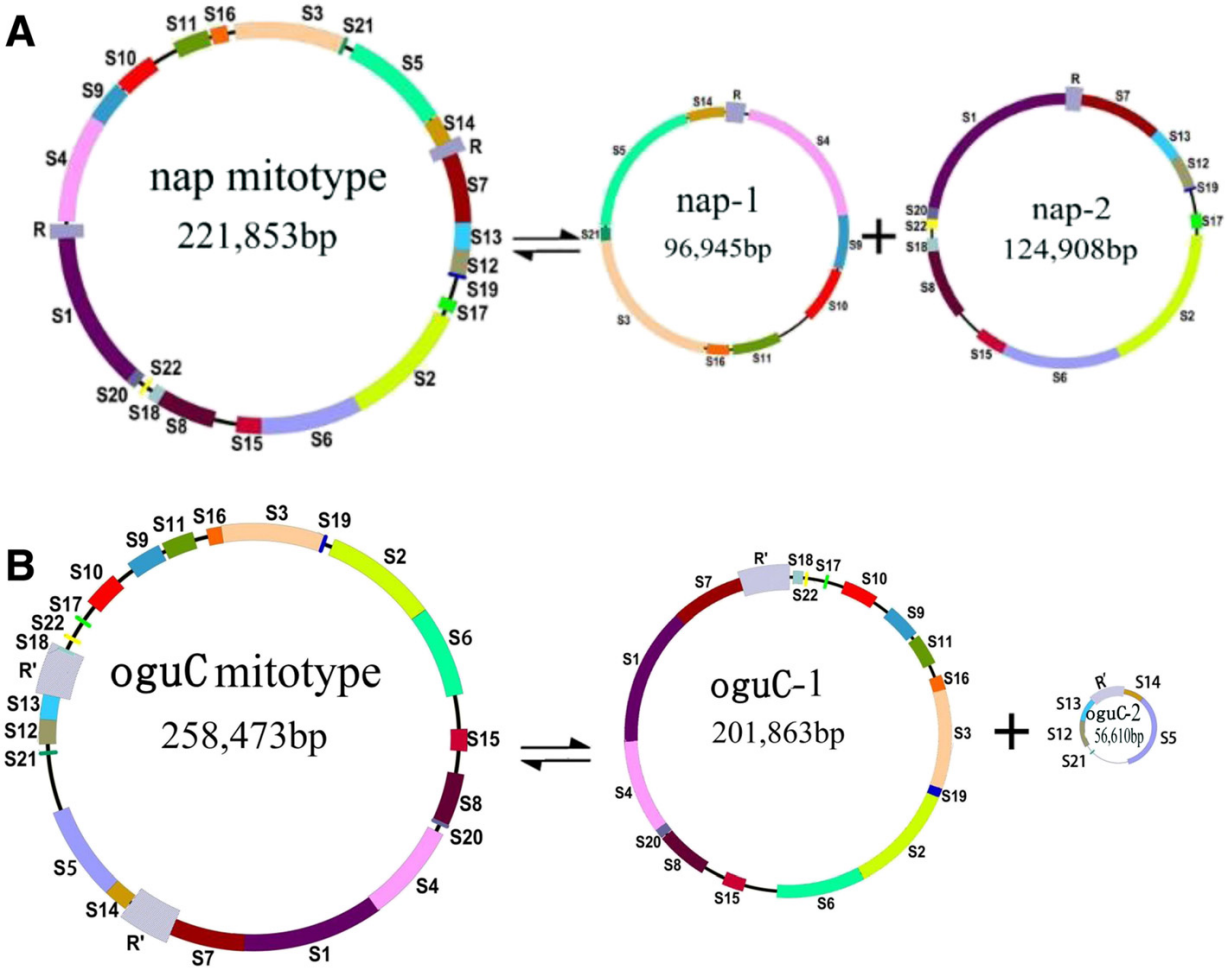
**The tripartite structure of the two mitotypes.** S1-S22 represent the syntenic region as illustrated in Figure 3. **A**, *oguC* mitotype; **B**, *nap* mitotype. R and R’, large repeat.

The large and short repeats were analyzed. The circle molecule had a pair of large repeat sequences (9731 bp) and only a *trn*Y gene was included. It is about four times the length of equivalent from *nap* (2427bp), but no sequence similarity was found between them (Figure 1). One of the two direct repeats in *oguC* occupied the non-syntenic region between S7 and S14 and another one extended from the end of S13 to the start of S18 (Figure 4). The presence of large repeats is believed to be associated with the formation of multipartite structure [15,32], which are isomeric forms that consist of the master circle and two smaller subgenomic circles (56610 bp and 201863 bp) via intramolecular homologous recombination in *oguC* (Figure 4). The coexistence phenomenon of various molecular forms was extensively predicted in many species sequenced that were verified through direct observation using electron microscopy in tobacco [33].

**Figure 5:**
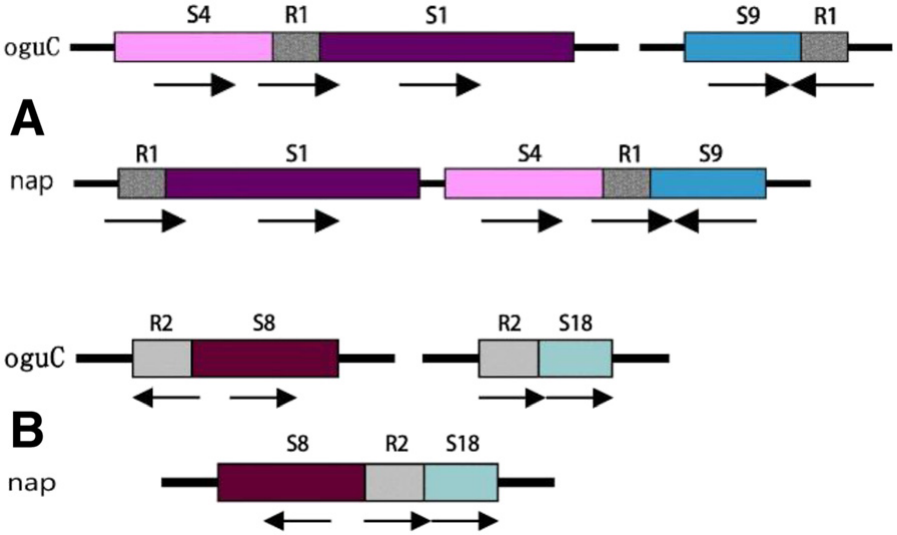
**The arrangement of syntenic regions caused by short repeats.** S1, 4, 9, 8,18 mean the syntenic region showed in Figure 3 and Figure 4. R1 and R2 represent short repeats. Arrow stands for the direction. **A** and **B**, the rearrangement induced by R1 and R2, respectively.

Apart from the large repeats, the mt genome of higher plants also distributes massive short repeats [25]. We identified 123 repeats (30–500 bp, similarity ≥ 90%), including both direct and inverted repeats, that were responsible for 6.54% of the genome. The short repeats contributed to genome reorganization and arrangements, although the frequency of these events was not as high [34,35]. We assayed reorganization relationship of some syntenic regions and found that two short repeats were closely related to the rearrangement of five syntenic domains, as elaborated on Figure 5. S1, S4, and S9 were originally located adjacent to each other in *nap*. S9 was on the opposite direction and only 610 bp was present between S1 and S4. However, because of the reorganization caused by the repeating R1 (310 bp), S4 and S1 transposed with each other that made the gap between them disappeared. On the other hand, S9 was separated to thousands of base pairs away and shifted the orientation. Another was the combined fragments of S8 and S19, which was divided by increasing the copies of R2 (232 bp) in *oguC*. Similar rearrangement relationship was also discovered in other mtDNA [15,17].

### Unique region of the genome

**Figure 6:**
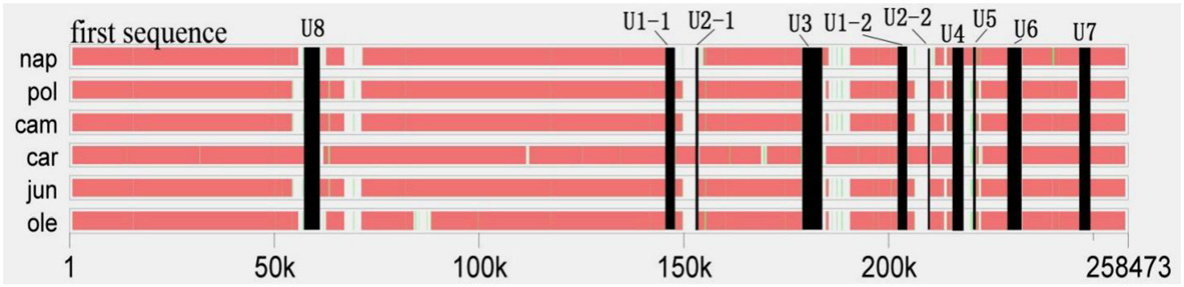
**The sequence alignment of 7 mtDNA of *Brassica *family.** The first sequence is just the invisible control mtDNA (*oguC*). The white and black regions represent the un-matched segment existing in every genome compared with *oguC* mt DNA. Only when the white region happens to emerge synchronously in other six mitotypes, this segment (black region) is considered to be the unique region.

We investigated the CMS-specific mitochondrial regions by comparing it with the other six entire mitochondrial genome sequences of *Brassica* species (Figure 6). Up to 11 unique regions, which constitute 8.89% of the genome, were assayed (Table 2). U1 and U2 had two identical copies that were included in the large repeats. U3 almost fully occupied the gap between S5 and S23, similar to U7, which possessed the non-syntenic region between S11 and S16. U3, which included *orf138*, must have come from the radish mtDNA. The speculation was proved again by the fact that this segment was almost 100% identical to the *orf138*-included region from *R. sativus* (accession number: Z18896). *Atp6* was equal to that from radish, with first 422 bp contained in the end of U7. A domain composed of U7 plus the next 862 bp of *atp*6 was also possibly from radish because a part of its sequence is found in radish (accession number: M24672). The other CMS-specific regions were included in the partial region of the non-syntenic domains. Analyzing those specific regions using Blastn searches against the NCBI database, with sizes ranging from 2% in U1 to 100% in U5, were aligned with the mtDNA of *Arabidopsis thaliana*. For the blast and non-blast region from specific region, we speculated that they were either the transfer of nuclear counterpart from one of the biparents or originated from the mtDNA of radish, but the latter hypothesis was more reasonable. More empirical proofs are needed to demonstrate this.

**Table 2:** **Unique region found in ***oguC*

**No.**	**Length**	**Fine location**	**Remark**	**ORF included**
U1-1	2220	145578-147797	included in the large repeats	
U1-2	2220	202186-204405		
U2-1	469	153028-153496		
U2-2	469	209635-210104		
U3	5130	178771-183898	*S5-S23	orf138
U4	1052	215584-216635	S22-S17	orf130
U5	445	220503-220947	S17-S10	
U6	3572	229073-232643	S10-S9	
U7	2700	246585-249284	* S11-S16	
U8	3828	57304-61131	S6-S15	orf122, orf102-1
U9	879	217471-218349	S22-S17	part of orf101-4

* Totally occupy the spacer between two syntenic regions.

### Homology with rapeseed chloroplast genome

Exogenous segments of the intergenic spacer are derived from the chloroplast genome and these sequences migrate and integrate into the mt genome [14,36]. Thus, we analyzed the homology between *oguC* mtDNA and rapeseed (*B. napus*) chloroplast genome. Six homologous segments with more than 95% identity were found in this genome (Table 3). The six segments ranged from 178 bp to 2196 bp, and accounted for 2.88% of the total mt genome. All of the sequences were located in the syntenic region.

**Table 3:** **Homologous segments to chloroplast of rapeseed found in *oguC *mitotype**

**No.**	**Fine location**	**Length**	**Identity**	**ORFs included**
H1	105622-107817	2196	97%	orf210, orf344-1
H2	8547-10426	1880	97%	orf100-2
H3	29583-30944	1362	96%	orf313
H4	193870-195028	1159	99%	orf257
H5	3828-4500	673	96%	orf170
H6	126258-126435	178	98%	

### ORFs and predicted CMS-related chimeric ORF in this genome

We detected 39 ORFs in this genome, with the shortest size equal to 303 bp, which summed to 7.41% of the mitotype. Of the 39 ORFs, 23 (similarity ≥ 99%) were shared in one or more *Brassica* genomes, which were remotely related to CMS. However, they are likely functional genes as these later-discovered genes *ccm*, *orf25* (*atp4*), and *orfB* (*atp8*) [37,38]. Of the remaining 16 unique ORFs in the *oguC* mitochondrial genome, 8 ORFs were totally not matched and 8 were partly identical to those present in the other six mitotypes. Five, including *orf138*, which is the *oguC*-related CMS gene, out of eight non-matched ORFs were completely or partially situated in the unique regions (Table 2). Three common ORFs and three unique ORFs were located in the cp-derived domains (Table 3). Among them, *orf210* and *orf344-1* were highly similar to 2 segments of the beta subunit of RNA polymerase, which were wholly situated in the chloroplast genome with a length of 1072 amino acids. When the intact nucleotide sequence of beta subunit of RNA polymerase was aligned with *oguC* mtDNA, H1 was found to be a truncated RNA polymerase beta subunit with 97% similarity. Some point mutations and indels resulted in the production of these two ORFs. Similar to *orf344-1*, a truncated ribulose-1, 5-bisphosphate carboxylase/oxygenase large subunit (*rbcL*) gene from cp genome evolved into *orf313*. Based on the three features of CMS related genes, namely, unique to the given mitotype, membrane-spanning domains and near to the functional genes [26,39], *orf138*, which encodes a 19 kDa transmembrane protein that showed toxicity to bacterial growth, can be suppressed by the nuclear *Rfo* locus [40-43].

## Discussion

**Figure 7:**
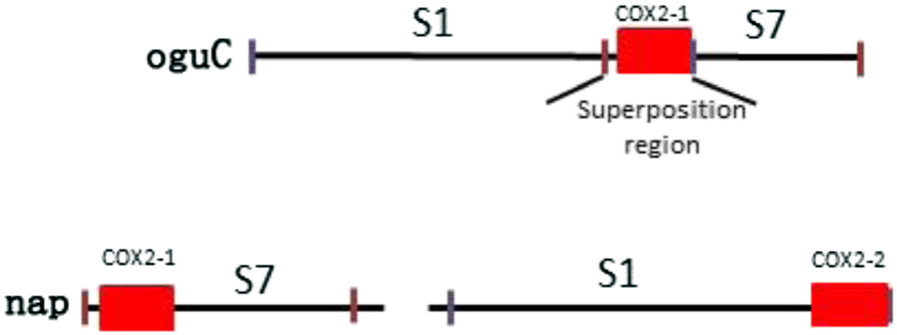
**The position of *cox *gene in *oguC *and *nap*.** S1 and S7 stand for the syntenic region as showed in Figure 2. Red box represents the *cox* gene.

We obtained the complete mt genome of the *oguC* devoid of *cox2-2* and increased copy of *atp9*. *Cox2-1* and *cox2-2* were distributed in the start region of S7 and the end region of S1 in *nap* mitotype (Figure 7), respectively. However, as the rearrangement and reconstruction of mt genome, S1 and S7 were translocated adjacent to each other in *oguC*. In addition, the first 2425 bp and last 2425 bp domains of S7, in which the *cox* gene was included, were incorporated into one superposition. Consequently, the *cox2-1* gene was saved whereas the *cox2-2* was lost. This phenomenon was also observed in *car* and *ole* mitotypes [17]. For the *atp9*, one was located in the syntenic region, which is in itself, and the other one was in the non-syntenic region. Compared to the NCBI data with a 767 bp segment, including the redundant *atp9* with its perimeter zone, we found that this segment was almost 100% identical to the corresponding section in the 940 bp domain from the mitochondrial *atp9* pseudogene of F0-F1 ATPase proteolipid from *R. sativus* (accession number: X69320). The complete 940 bp segment from radish was likely broken into two parts; one with *atp9* included was fused into the reconstructed mitochondria during the rearrangement and reorganization that resulted from the collision of the cytoplasm of two cells, and the other one was lost. Additionally, *tatC* and *atp6* were also reasonably speculated that they were fused from radish based on the additive length absent from the other 6 lines of *Brassica* family while completely identical to the counterpart of radish (Figure 2). However, although *cox1*, *rps12*, *atp8* and *ccmC* share completely or partly same nucleotide sequence between *oguC* and radish (Table 1), we still can’t make sure whether they were invaded from radish or not only by the SNPs analysis.

When *nap* mtDNA was taken as the control, 22 syntenic regions were detected in total. Estimating the number of recombination events was difficult because of the many syntenic regions. However, relative to two more similar mitotypes (*pol* and *nap*) that have 13 syntenic region with the same analysis criterion (length ≥ 1000 bp, similarity ≥ 95%) [15], it showed complex reconstruction. *oguC* mitotype must have undergone complicated changes and evolutionary events when the cytoplasm of two cells contacted each other.

The large repeat (R’) in *oguC* was longer than others, except for R1, which mediates the homologous recombination with another two repeats, R and R2 in *ole*. The 5109 bp segment of R’ showed 99% similarity with R3 in *car*. Interestingly, except for *ole*, the six mt mitotypes contained only one large repeat, four of which contained the same large repeat, R (Table 4).

**Table 4:** The composition of mitochondrial genomes of 7 mitotypes from *Brassica *family*

**Feature**		***oguC***	***nap***	***pol***	***cam***	***car***	***jun***	***ole***		
Accession number		-	AP006444	FR715249	JF920285	JF920287	JF920288	JF920286		
Genome size (bp)		258473	221853	223412	219747	232241	219766	360271		
GC%		45.21	45.22	45.19	45.24	45.33	45.24	45.20		
Gene No.	Protein-coding genes	33	35	34	34	33	34	56		
	tRNA	23	17	18	18	17	18	35		
	rRNA	3	3	3	3	3	3	4		
	total	59	55	55	54	53	54	95		
	%	16.48	17.45	17.34	17.35	15.82	17.35	17.68		
ORF	ORFs	40	46	45	44	36	44	44		
Unique region (%)		8.60	0.74	0.28	0	1.57	0	0		
cp-derived sequences (%)		2.88	3.36	3.33	3.39	3.21	3.39	3.53		
Large repeat (bp)		R’	R	R	R	R3	R	R	R1	R2
		9713	2427	2427	2427	6580	2427	2427	141800	3605
Short repeat (%)		6.54	7.13	6.57	6.34	6.29	8.70	5.31		

* Part of the data cited from [15,17].

Using one genome of the seven mitotypes as the control to find the unique regions for every mitotype, the percentage ranged from 0% in three mitotypes (*cam*, *jun*, and *ole*) to 8.60% in *oguC*. Both *nap* and *car* contained three shorter specific segments constituting 0.74% and 1.57% of these two genomes, respectively. A 620 bp unique segment located in *pol* contributed 0.28% to its total genome. In terms of the percentage, at least 7% of the exogenous sequences from radish mtDNA coexist with the *oguC* mtDNA (Table 4). When searched against the NCBI databases using those specific segments, similar alignments to that of *oguC* were obtained, some of which resembled those in *Arabidopsis thaliana*.

We also predicted the cp-derived sequence, which was intriguing because the seven mitotypes were blasted for the identical six segments with identities more than 95% (Table 4). However, because of the large copy of R1, five cp-derived segments had two copies in *ole*. From the cp-derived data, we found that *Brassica* species have stable sources of chloroplast sequence.

## Conclusions

This study finished mtDNA sequencing of a *Ogura*-cms-cybrid (*oguC*), which derived from somatic fusion between *Brassica napus* and sterile radish. By contrast to one or more of six other *Brassica* lines, we reasonably speculated that *tatC* gene and 2 unique regions, U3 and U7, must be introgressed from radish. In addition, the rearrangement mediated by large and short repeats between these two parental mtDNAs extensively existed. With regard to the evolution of this integrated CMS mtDNA, more data need to be known.

## Methods

### Plant material and mitochondrial genome extraction

Seed of *Brassica napus* (*ogu*-CMS cybrid, *oguC*) was kindly provided by Norddeutsche Pflanzenzucht, Hans-Georg Lembke KG (Germany). The etiolated one-week-old *Brassica napus* seedlings were prepared; the mitochondria and mtDNA extraction were performed following previously published methods [15]. To satisfy the requirements for 454 sequences, the minimum criterion for sample concentration was 50 ng/μl and total amount was equal to at least 20 μg.

### Genome sequencing

A shotgun library that includes short and long paired end libraries were constructed simultaneously, which was followed by emulsion-based clonal amplification (emPCR) for DNA library bead enrichment. Finally, a genome sequencer FLX operation was conducted and the system output was derived. The contigs were joined by PCR sequencing. For the *oguC* genome, high quality read number, high quality bases, average read length, and sequencing depth were 8387, 3,913,351 bp, 469.3 bp, 15.2X, respectively. For SNP analysis and unique regions in *oguC* resequence was done.

### Genome analysis

The genes scattered in this genome were annotated using the Blast service of NCBI. tRNAscan [44] and ORF finder (http://www.ncbi.nlm.nih.gov/gorf/gorf.html) were used to identify the tRNA sequences and potential ORFs, respectively. The unique regions of seven genomes were dug out with MultiPipMaker [45]. BlastN was used to discover large repeats. Short repeats were detected using commercial software developed by Shanghai Majorbio Bio-pharm Biotechnology Company (China). The accession numbers of the mtDNA are listed in Table 4.

## Competing interests

The authors declare that they have no competing interests.

## Authors’ contributions

YW and RG conceived and designed the study. JW, JJ, AL participated in the experiments. JW, XL and YZ analyzed the data. All authors drafted the manuscript and approved the final manuscript.
